# Cerebral autoregulation in the hyperacute phase after experimental subarachnoid haemorrhage using an endovascular perforation model

**DOI:** 10.1093/braincomms/fcag356

**Published:** 2026-09-16

**Authors:** Michael Veldeman, Erta Beqiri, Marek Czosnyka, Mark Coburn, Anke Höllig, Peter Smielewski

**Affiliations:** Department of Neurosurgery, Rheinisch-Westfälische Technische Hochschule, Aachen University Hospital, 52074 Aachen, Germany; Brain Physics Laboratory, Division of Neurosurgery, Department of Clinical Neurosciences, University of Cambridge, Hills Rd, Cambridge CB2 0QQ. UK; Brain Physics Laboratory, Division of Neurosurgery, Department of Clinical Neurosciences, University of Cambridge, Hills Rd, Cambridge CB2 0QQ. UK; Brain Physics Laboratory, Division of Neurosurgery, Department of Clinical Neurosciences, University of Cambridge, Hills Rd, Cambridge CB2 0QQ. UK; Department of Anesthesiology and Intensive Care Medicine, University Hospital Bonn, 53127 Bonn, Germany; Department of Neurosurgery, Rheinisch-Westfälische Technische Hochschule, Aachen University Hospital, 52074 Aachen, Germany; Brain Physics Laboratory, Division of Neurosurgery, Department of Clinical Neurosciences, University of Cambridge, Hills Rd, Cambridge CB2 0QQ. UK

**Keywords:** subarachnoid haemorrhage, rat, cerebral autoregulation, pressure reactivity index, flow reactivity index

## Abstract

Impaired cerebral blood flow contributes to delayed neurological deficits after subarachnoid haemorrhage, but cerebral autoregulation, which regulates cerebral blood flow to the brain in the hyperacute phase is poorly defined. We characterized early pressure- and flow-based reactivity in a rat subarachnoid haemorrhage perforation model. This is a retrospective analysis of older data acquired to investigate a different hypothesis. Male Sprague–Dawley rats underwent endovascular perforation subarachnoid haemorrhage. Arterial blood pressure, intracranial pressure and bilateral laser Doppler flow were continuously recorded. Pressure reactivity (PRx*) and flow reactivity (LDx*) were calculated as 5-min moving Pearson correlations between arterial and intracranial pressure and arterial pressure and laser Doppler flowmetry, respectively. Exposure above impairment thresholds (PRx* > 0.3; LDx* > 0.5) was compared pre-subarachnoid haemorrahge (−30–0 min) versus post-subarachnoid haemorrhage (15–60 min) and related to haemorrhage severity. Group-mean PRx* and LDx* remained below impairment thresholds. Nevertheless, time with PRx* > 0.3 increased after subarachnoid haemorrhage (median 0.0% versus 1.9%, *P* < 0.001) and correlated with intracranial pressure elevation (*r* = 0.527, *P* < 0.001). LDx* showed no significant pre–post change in either hemisphere. PRx* and LDx* correlated weakly (*r* = 0.089, *P* < 0.001) with frequent discordance. Cerebral autoregulation is largely preserved within 60 min after subarachnoid haemorrhage, with transient pressure–reactivity impairment in a subset linked to intracranial pressure peaks. Pressure- and flow-based indices provide non-interchangeable information.

## Teaser

Veldeman *et al*. report that cerebral autoregulation, assessed using pressure and flow-based correlation indices, is largely preserved within the first hour following experimental subarachnoid haemorrhage in rats. Transient impairment of pressure reactivity occurs in a subset of animals and is proportional to the magnitude of intracranial pressure elevation. Pressure- and flow-based indices provide non-interchangeable information.

## Graphical Abstract

**Graphical Abstract:**
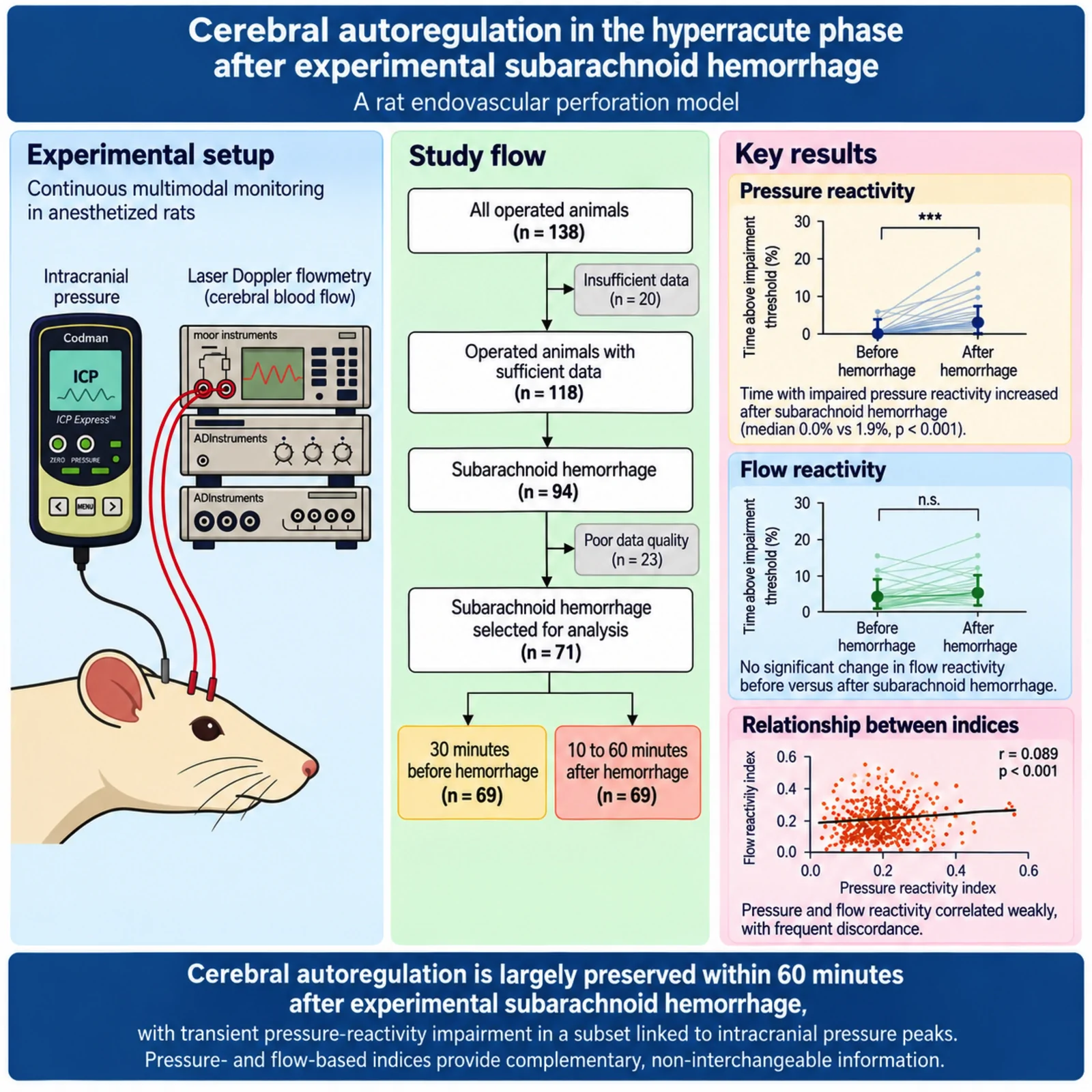

## Introduction

Aneurysmal subarachnoid haemorrhage (SAH) affects approximately 7–10 per 100 000 individuals annually and remains among the most devastating forms of stroke.^1,2^ Case fatality rates can exceed up to 30% and there exists substantial morbidity among survivors.^3,4^ Despite advances in neurocritical care and endovascular interventions, SAH management remains difficult due to the complex cascade of pathophysiological events following aneurysm rupture.

The immediate aftermath of rupture is characterized by early brain injury, involving acute elevations in intracranial pressure (ICP), global cerebral ischaemia and disruption of the blood–brain barrier.^5,6^ The abrupt extravasation of arterial blood into the subarachnoid space causes a dramatic rise in ICP, equalling or exceeding systemic arterial pressure, leading to a transient global ischaemia. This primary insult initiates a cascade of secondary injury mechanisms that evolve over time.

Dysfunction of cerebral autoregulation is identified as a factor contributing to delayed cerebral ischaemia (DCI).^7-9^ Under normal conditions, autoregulation ensures adequate cerebral blood flow through dynamic modulation of cerebrovascular resistance. Following SAH, this mechanism is frequently impaired, increasing the brain’s vulnerability to ischaemic and hyperaemic injury.^10^

Cerebral blood flow regulation encompasses several complementary mechanisms across different spatial and temporal scales. Among those, the myogenic response (autoregulation) regulates vessel tone based on transmural pressure changes, while metabolic mechanisms respond to local tissue oxygen and CO_2_ levels.^11^

At the bedside, cerebral autoregulation can be assessed via the pressure reactivity index (PRx), which quantifies the relationship between slow fluctuations in arterial pressure (ABP) and ICP.^12,13^ A positive PRx suggests impaired autoregulation, while mildly negative values indicate preserved cerebral autoregulation. Laser Doppler flowmetry (LDF) uses Doppler-shifted light signals via subdural probes to calculate cortical blood flow as a relative measure of regional cerebral blood flow (rCBF).^14^ The LDx can be calculated as a moving correlation coefficient between ABP and LDF, which may provide additional insight into autoregulatory status at a local microvascular level.

Little is known about cerebral autoregulation in the hyperacute phase immediately following aneurysm rupture and whether this affects the further course of disease. Clinically, patients typically present hours after symptom onset, and placement of neuromonitoring is often delayed until after aneurysm securing and stabilization.^15^

### Objectives

This study aims to characterize cerebral autoregulation in the hyperacute phase following experimental SAH using both pressure-based (PRx) and flow-based (LDx) indices. Objectives are to (i) assess temporal changes in reactivity immediately after SAH induction, (ii) examine the relationship between haemorrhage severity and potential autoregulatory impairment and (iii) evaluate whether pressure- and flow-based indices offer complementary or redundant information on early autoregulatory function.

## Material and methods

### Study design and ethical approval

This post hoc analysis was based on data collected as part of experiments investigating the neuroprotective effects of argon, in a rat model of SAH.^16^ All procedures involving animals were approved by the responsible governmental authority (protocol number TVA 10416G1, Landesamt für Natur, Umwelt und Verbraucherschutz NRW, Recklinghausen, Germany) and conducted in accordance with the Guide for the Care and Use of Laboratory Animals (8th Edition, National Research Council, United States). Experimental reporting follows the ARRIVE guidelines (Animal Research: Reporting of In Vivo Experiments).^17^

### Animals and group allocation

Male Sprague-Dawley rats (Charles River, Sulzfeld, Germany) weighing between 300 and 400 g (Charles River, Sulzfeld, Germany) at the time of surgery were housed under standard conditions, including a 12-h light–dark cycle and ad libitum access to food and water. Animals were acclimated for a minimum of one week prior to the procedure. Randomization into SAH and sham groups was done by drawing lots. In the original study design, a subset of animals was treated with 50% argon ventilation beginning 1 h after SAH or sham surgery.^16^ For the purpose of this analysis, only data acquired prior to the initiation of any treatment were used.

### Anaesthesia, monitoring and postoperative care

Anaesthesia was induced by intraperitoneal injection of midazolam at 2 mg/kg (Ratiopharm, Ulm, Germany), medetomidine at 0.15 mg/kg (Zoetis, Florham Park, NJ) and fentanyl at 0.0075 mg/kg (Rotexmedica, Trittau, Germany). To ensure consistent anaesthetic depth, one-quarter of the induction dose was administered subcutaneously every 30–45 min. Animals breathed spontaneously through a face mask. Adequacy of ventilation was confirmed by repeated blood gas analyses, performed at least once per hour.

An arterial catheter was inserted into the tail artery for continuous invasive blood pressure monitoring and intermittent blood gas and electrolyte measurement. Core body temperature was maintained at 37°C throughout surgery and the postintervention observation period using a feedback-controlled heating pad (Physitemp Instruments, Clifton, NJ). Postoperative analgesia was provided by intramuscular administration of metamizole at 20 mg/kg every 8 h. At the conclusion of the experiment, euthanasia was performed by exsanguination under deep anaesthesia.

### Surgical procedure and data acquisition

This study used an established model of endovascular perforation to induce SAH. Regional cerebral blood flow (rCBF) within the middle cerebral artery territory was continuously monitored using bilateral laser Doppler flowmeter (LDF; Moor Instruments, Axminster, UK). With the animal in a prone position, a midline scalp incision was made and the skull was exposed. The bregma was identified, and the bone was thinned bilaterally 5.5 mm lateral and 1 mm posterior to bregma using a water-cooled high-speed drill. LDF probes were affixed to these prepared sites.

Intracranial pressure (ICP) was monitored through a microsensor probe (Codman ICP Express; Codman/DePuy, Raynham, MA), inserted via a small craniotomy over the parietal cortex. SAH was induced using two variations of the perforation technique. The classical approach employed a 3-0 polypropylene suture (Prolene; Ethicon, Somerville, NJ) with a diameter of 200–250 µm. The modified method involved a polytetrafluoroethylene tube (outer diameter 400 µm, inner diameter 200 µm; VWR, Radnor, PA) containing a tungsten wire with a diameter of 80 µm (Salomons Metalen, Groningen, NL).

After surgical exposure of the left common and internal carotid arteries, the filament or wire was advanced until vessel perforation was achieved. SAH induction was verified by a rapid increase in ICP and a simultaneous bilateral reduction in rCBF. Following successful induction, the perforation tool was withdrawn and the carotid artery ligated. Sham-operated animals underwent the same surgical preparation without perforation of the vessel.

**Figure 1:**
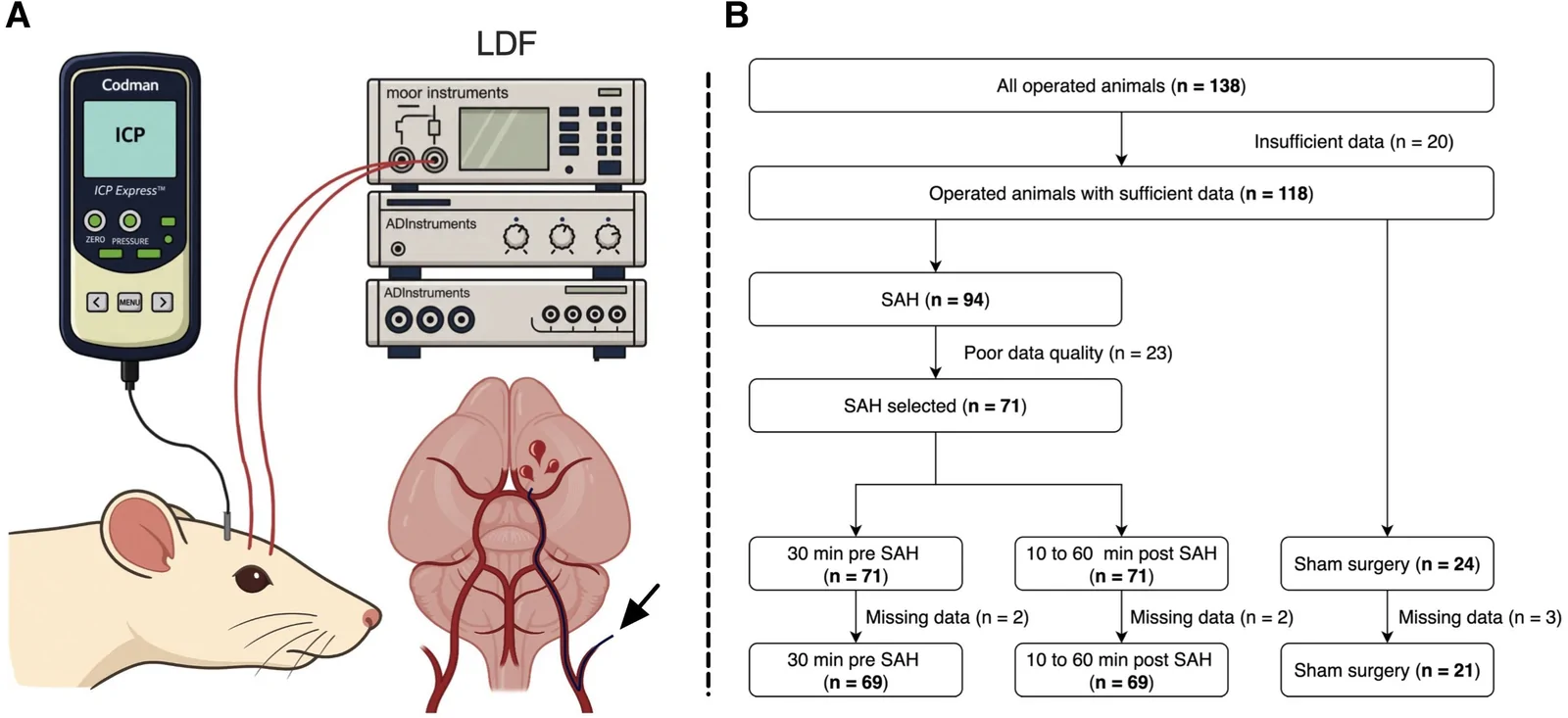
**Experimental set up and animal inclusion flow chart.** (A) Design of the experimental set up. Subarachnoid haemorrhage (SAH) was induced via endovascular perforation of the anterior-middle cerebral artery bifurcation using a filament or wire introduced via the external and then through the internal carotid artery (arrow). Regional cerebral blood flow (rCBF) was monitored bilaterally via laser Doppler flowmetry (LDF) probes over the thinned skull, while intracranial pressure (ICP) was measured with a microsensor probe. A sharp ICP rise with bilateral rCBF drop confirmed successful SAH. Arterial blood pressure (ABP) was registered via an intra-arterial catheter in the tail artery (not depicted). Physiological parameters were recorded continuously at 250 Hz. **(B)** Flow chart of animal inclusion. From all SAH animals, 25 were excluded due to poor quality of or insufficient data. Three sham animals were excluded due to insufficient available ABP data. Assessment of the optimal time window for cerebral autoregulation index calculation was done in a cohort of 21 sham animals. These windows were transferred to the SAH cohort consisting of 69 animals.

Physiological parameters, including ABP, ICP, laser Doppler flow (LDF), were continuously recorded using a PowerLab data acquisition system (ADInstruments, Spechbach, Germany) at a sampling rate of 250 Hz, and ECG at a sampling rate of 1000 Hz. The experimental setup is illustrated in Fig. 1A.

After euthanasia, the base of the brain was inspected, and the amount of subarachnoid blood scored on an ordinal scale between 0–18, as described by Sugawara *et al*.^18^

### Data processing

After acquisition, physiological time series data were imported into ICM+® multimodal monitoring software (University of Cambridge, Cambridge Enterprise, Cambridge, UK).^19^

Sections of ABP with loss of amplitude due to clogging of the arterial catheter were omitted after visual inspection. In addition, ABP and ICP signals were cleaned with automated methods via thresholding for physiological plausibility (ABP values below −30 and above 300 were removed, ICP values below −30 and above 200 were removed), and reliable amplitude detection (ABP sections with amplitude below 2.66 and above 100 were removed, as per empirical distribution thresholding). Animals with insufficient data quality (<30% ABP and <30% bilateral LDF completeness after artefact removal) were excluded. Visualization of power spectra density charts of ABP and ICP on 21 sham operated animals allowed to identify suitable frequency ranges for assessment of slow wave activity. The respiratory rate was identified at 0.4 Hz and the heart rate at 5–6 Hz, which was consistent with previous literature.^20,21^ Based on these observations, ABP, ICP and LDF measurements were averaged over 2.5-second interval, updated every second. This moving averaging functions as a low-pass filter that excludes the arterial pulse and respiratory rate. To remove the influence of the step change in ICP after SAH induction, each recording was split into two periods: baseline (pre- step change) and after SAH induction (starting from the rise in ICP). The 15 min following the SAH induction were not considered in the following autoregulation-based analysis (referred to as the ‘post’ period). To remove the effect of the Cushing response after SAH induction and of baseline jumps in ABP due to catheter clogging, detrending was applied to ABP, ICP and LDF signals at 1 Hz resolution, to the pre- and post-periods separately, after data cleaning. This was achieved with a high pass filter with elliptic design, cut-off frequency 0.016 Hz, transition bands 0.001–0.05 Hz, stopband attenuation of 60 dB, passband ripple of 0.001. The edge effect of the filter was removed by subtracting 110 data point at the beginning of each continuous period of recorded data.

Mean arterial pressure (MAP) was calculated and cerebral perfusion pressure (CPP) was determined as MAP *minus* ICP.

Assessment of cerebral autoregulation was intended based on the pressure reactivity index (PRx)^12^ and ABP-LDF correlation (LDx). A correlation window of 30 (including 30 data points), 60, 120 and 300 s was explored to calculate both reactivity indices. The 300 s window was chosen for further analyses based on confirmation of corresponding time profiles between the different options, with the 300 s window showing less variance (Suppl. Figs 1 and 2). The stability of CPP was also tested across the different time windows (Suppl. Fig. 3). The variation of CPP standard deviation across 30 s, 1 and 5 min sliding windows were calculated. Shorter windows revealed greater variability due to transient fluctuations. However, at 5-min resolution, the CPP signal demonstrated low variation in standard deviation (mean |Δ Std| < 0.03 mmHg). This supports the assumption of the stability of the physiological state. The window update time was set at 1 s. Second by second time trends were considered for all variables.

Given the settings for calculating PRx and LDx were adapted as described above and tailored to this animal experimental study, the adjusted indices are therefore referred to as PR* and LDx*, in the rest of this text.

LDF data were corrected to individual animal baselines by calculating the mean LDF value during a 5-min window immediately preceding SAH induction (−300 to 0 s) for each hemisphere separately. Baseline-adjusted LDF values were computed by subtracting the individual baseline mean from all raw LDF measurements.

### Temporal analysis of cerebral autoregulation

To assess the temporal evolution and impact of SAH on cerebrovascular autoregulation the percentage of animals with impaired autoregulation was calculated at each timepoint defined by PRx* > 0.3 and LDx* > 0.5, using 1-min time bins. The PRx threshold of 0.3 was chosen as it previously demonstrated significant discriminatory power for worse outcome in patients with traumatic brain injury.^22,23^The cut-off for LDx was adopted from previous research in an experimental TBI setting.^24,25^

The analysis focused on two distinct periods: pre-SAH baseline (−30 to 0 min) and post-SAH response (15–60 min), with the immediate post-SAH period (0–15 min) excluded due to acute physiological instability as a result of the step change in ICP and Cushing reflex.

Pre-SAH exposure was calculated from the 30-min baseline period, while post-SAH exposure was derived from the 45-min period following the acute phase.

### Cerebral autoregulation in relation to haemorrhage severity

To investigate whether haemorrhage severity correlates with the degree of autoregulation dysfunction, we analysed the relationship between SAH severity markers and autoregulation impairment metrics. Severity assessment was performed using three markers: (i) ICP increase, defined as the peak intracranial pressure rise following SAH induction; (ii) LDF left drop, representing the reduction in laser Doppler flowmetry perfusion units on the SAH-affected hemisphere from baseline to minimum post-SAH value and (iii) Sugawara grading, an ordinal scale (0–18) quantifying subarachnoid blood volume where higher scores indicate more severe haemorrhage.^18^

Autoregulation impairment was quantified using the proportion of time spent above the prespecified thresholds, calculated for the post-SAH analysis period (15–60 min).

### Data analysis and graphics

All statistical analyses were performed using Python version 3.13 with the following libraries: pandas, numpy, matplotlib, seaborn, scipy and scikit-learn.

Continuous variables are presented as median with interquartile range (IQR) due to non-normal data distributions, as determined by visual inspection and Jarque-Bera normality testing. Categorical variables are presented as frequencies and percentages.

Pre-to-post SAH comparisons of the percentage of time spent above impairment thresholds (PRx* > 0.3 and LDx* > 0.5) were performed using the Wilcoxon signed-rank test for paired non-parametric data, comparing the pre-SAH baseline period (−30 to 0 min) with the post-SAH analysis period (15–60 min). The rank-biserial correlation was reported as effect size, with 95% confidence intervals derived by bootstrap resampling (5000 iterations).

Relationships between SAH severity markers (ICP increase, LDF drop, Sugawara grading) and autoregulation impairment metrics (percentage of time with PRx* > 0.3 and LDx* > 0.5 during the post-SAH period) were assessed using Pearson correlation analysis with linear regression modelling. Regression lines were styled according to statistical significance (solid: *P* < 0.05, dashed: *P* < 0.1, dotted: *P* ≥ 0.1). Pearson correlations are reported with 95% confidence intervals derived from the Fisher z-transformation.

Agreement between PRx* and LDx* categorical classifications was assessed during the post-SAH period (15–60 min) using overall percentage agreement and Cohen's kappa coefficient, calculated across all paired second-by-second observations. The overall correlation between PRx* and LDx* time series was quantified using Pearson correlation across all paired observations, reported with 95% confidence intervals derived from the Fisher z-transformation.

Animals with insufficient data (less than 5 min of valid paired data) were excluded from relevant analyses. Statistical significance was set at *P* < 0.05 for all tests.

## Results

### Animals

**Figure 2:**
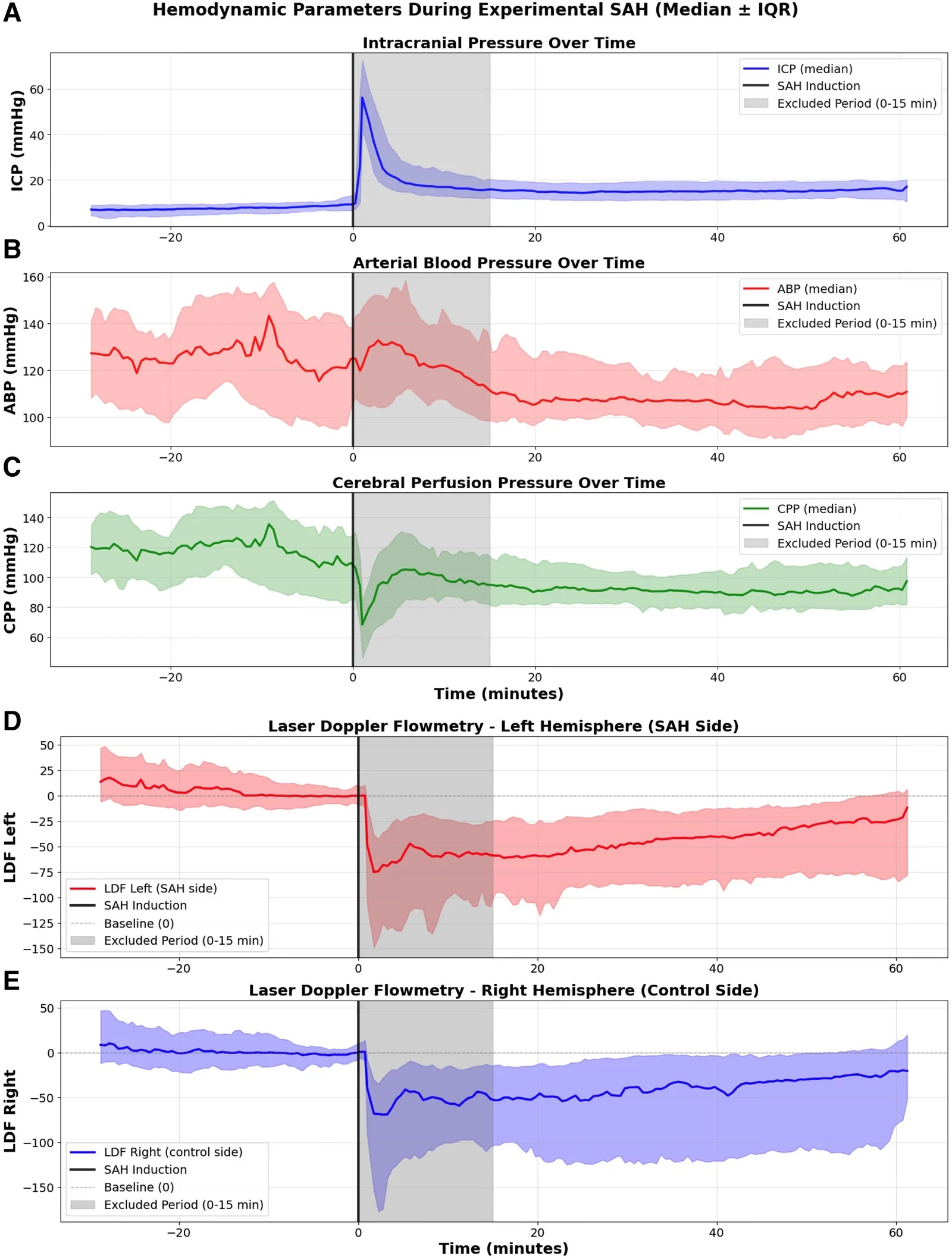
**Temporal evolution of ICP, ABP, CPP and left and right LDF during experimental subarachnoid haemorrhage (SAH).** Data represent median values with interquartile ranges (IQR, shaded areas) from 69 animals. Time 0 indicates the moment of SAH induction, with 30 min of baseline data preceding induction and 60 min of post-SAH monitoring. Data are binned in 30-s intervals. **(A)** Intracranial pressure (ICP, blue) shows stable baseline values (∼8 mmHg) pre-SAH, followed by a characteristic acute spike to ∼60 mmHg immediately upon SAH induction, then gradual decline to elevated steady-state levels (∼17 mmHg). **(B)** Arterial blood pressure (ABP, red) demonstrates relatively stable pre-SAH values (∼127 mmHg) with an elevation following SAH induction as the result of a Cushing reflex. This increase is followed by progressive decline to lower sustained levels (∼108 mmHg) post-SAH. **(C)** Cerebral perfusion pressure (CPP, green) shows stable pre-SAH values (∼120 mmHg) followed by an acute drop to ∼65 mmHg immediately post-SAH and partial recovery to sustained levels (∼90 mmHg). **(D)** Left and **E** right hemisphere laser Doppler flowmetry (LDF Left, red; LDF Right, blue) demonstrates the ipsilateral response to SAH induction. Absolute values have been corrected for baseline difference by subtracting the mean value over a 5-min time window preceding SAH induction. Flow shows an immediate and sustained reduction following SAH, and remains persistently reduced throughout the 60 min after SAH induction.

After manual inspection and cleaning of ABP, ICP and LDF signals, 69 SAH animals and 21 sham animals were identified with sufficient individual and overlapping (ABP-ICP and ABP-LDF) data for inclusion. An inclusion flow chart is presented as Fig. 1B. The course of ICP, APB, CPP and LDF from the left and right hemisphere is plotted overtime, before and after induction of SAH in Fig. 2.

### Cerebral autoregulation after experimental SAH

**Figure 3:**
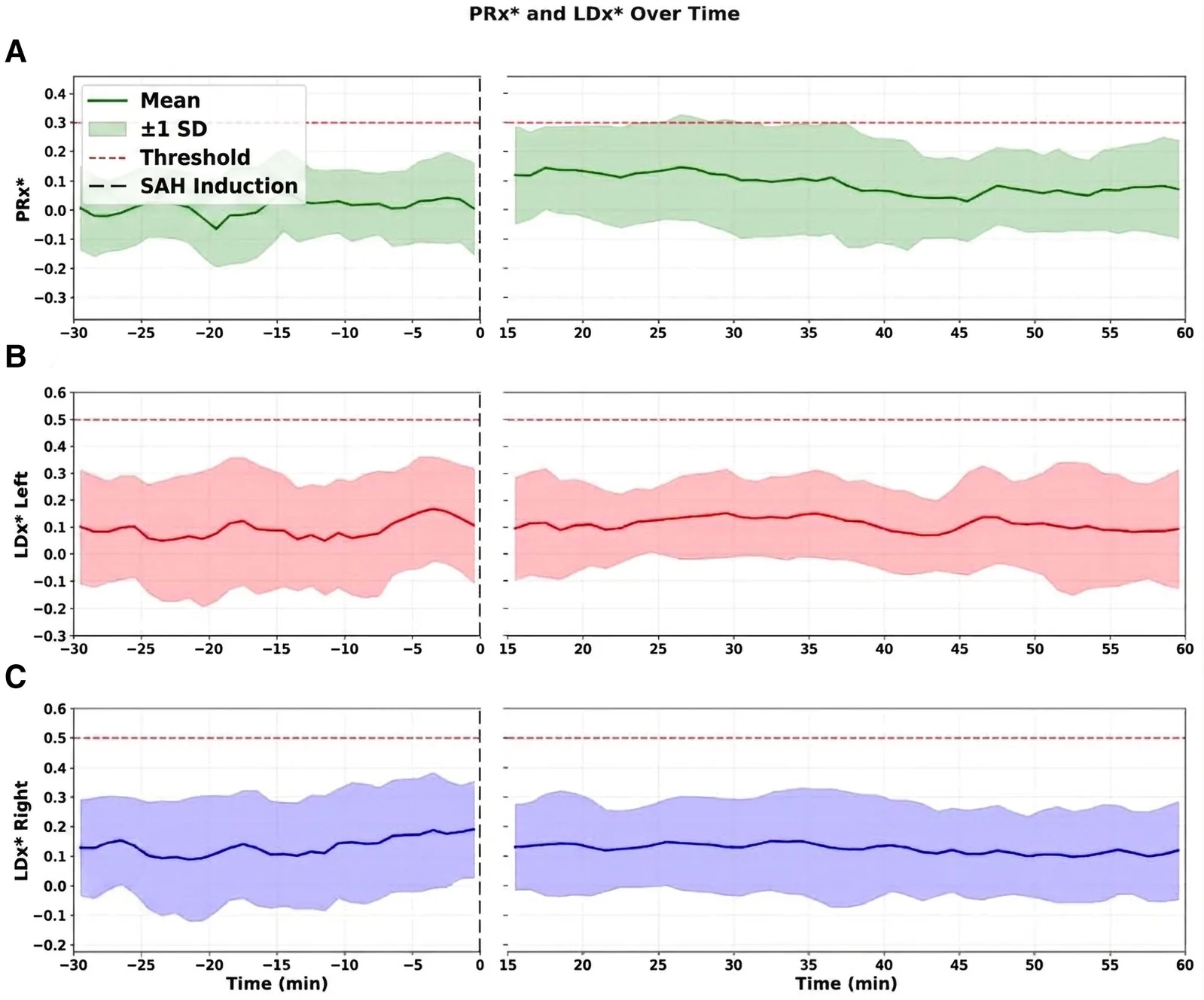
Course of ABP-ICP based pressure reactivity index (PRx*) and ABP-LDF based flow reactivity index (LDx*) before and after induction of subarachnoid haemorrhage (time point 0). The x-axis is broken, omitting the first 15 min of data directly after haemorrhage induction. (**A**) Pressure reactivity index (PRx*) values remain near zero before SAH induction (intact autoregulation). Values of PRx* continuously remain below the 0.3 impairment threshold. The number of data points contributing to each 1-min bin ranged from 1348 to 1966 pre-SAH and 1932 to 2570 post-SAH. (**B**) Left hemisphere LDx* index remains relatively stable around 0.1 throughout the observation period, staying below the impairment threshold (0.5) both pre- and post-SAH. The number of data points contributing to each 1-min bin ranged from 1743 to 2208 pre-SAH and 2279 to 2752 post-SAH. (**C**) Right hemisphere LDx* index behaves similarly to the left hemisphere; values remain stable near 0.1 and below the impairment threshold (0.5). The number of data points contributing to each 1-min bin ranged from 1743 to 2208 pre-SAH and 2279 to 2752 post-SAH.

Population-level analysis of cerebral autoregulation revealed no evidence of impaired autoregulation during the acute phase following experimental SAH (Fig. 3). Group-averaged indices for PRx* and LDx* remained below established impairment thresholds, despite some inter-individual variability.

Although confidence intervals around the group means were moderately wide, particularly for PRx*, these did not cross the impairment threshold in a sustained manner. Summary statistics of physiological data and derived metrics for the pre and post periods are provided in Suppl. Table 1. An analysis of ABP and ICP variability within the 5-min PRx* calculation windows is presented in Suppl. Fig. 4.

PRx* values remained stable throughout the observation period, with mean values consistently below the 0.3 impairment threshold in both pre- and post-SAH phases (Fig. 3A). No distinct time trend was observed, and PRx* trends showed only modest fluctuation over time.

LDx* values from both the left (SAH-affected) and right (contralateral) hemispheres also remained below 0.5 throughout (Fig. 3B and C). Mean LDx* levels hovered between 0.05 and 0.15, with no marked divergence between hemispheres or across time.

**Figure 4:**
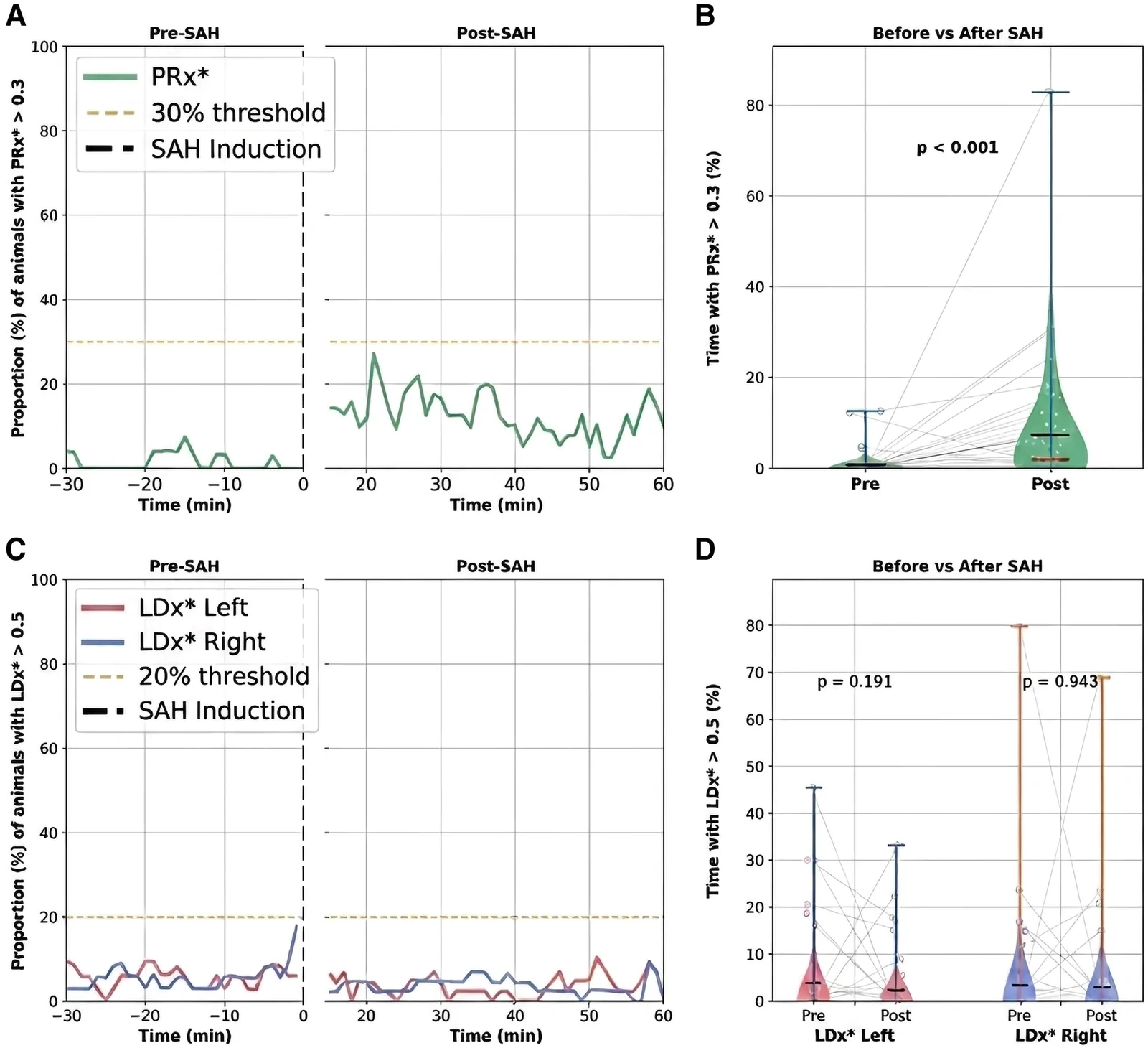
Temporal analysis of autoregulation impairment based on PRx* > 0.3 and LDx* > 0.5. (A) Timeline analysis showing the proportion of animals with PRx* > 0.3 over time (green line). The orange dashed line indicates an arbitrary 30% reference threshold. The number of animals contributing to each timepoint ranged from 27 to 69 (out of 69 total animals), as some timepoints did not contain valid PRx* data for every animal. The black dashed line marks the SAH induction timepoint. Throughout the post-SAH period, the proportion of animals with PRx* > 0.3 does not exceed 30%. **(B)** Before-after comparison of time spent with PRx* > 0.3 (expressed as percentage of the analysed period), comparing the 30-min pre-SAH window to the 15–60-min post-SAH window. Violin plots show median (red horizontal line), mean (black horizontal line), range (vertical line with caps) and individual animal data points (circles). Wilcoxon signed-rank test for paired data (one value per animal per window): pre-SAH median 0.00% [IQR 0.00–0.00%] versus post-SAH median 1.88% [IQR 0.00–8.56%]; *W* = 45.0, *P* < 0.001, *n* = 45. **(C)** Timeline analysis showing the proportion of animals with LDx* > 0.5 from the left (SAH-affected) hemisphere (red line) and right (contralateral) hemisphere (blue line). The orange dashed line indicates an arbitrary 20% reference threshold. The number of animals contributing to each timepoint ranged from 27 to 69 (out of 69 total animals), as some timepoints did not contain valid LDx* data for every animal. The black dashed line marks the SAH induction timepoint. Throughout the post-SAH period, the proportion of animals with LDx* > 0.5 does not exceed 20%. **(D)** Before-after comparison of time spent with LDx* > 0.5 (expressed as percentage of the analysed period), comparing the 30-min pre-SAH window to the 15–60-min post-SAH window. Violin plots show median (red horizontal line), mean (black horizontal line), range (vertical line with caps) and individual animal data points (circles). Wilcoxon signed-rank test for paired data (one value per animal per window): LDx* Left – pre-SAH median 0.00% [IQR 0.00–0.62%] versus post-SAH median 0.00% [IQR 0.00–0.00%], *W* = 70.0, *P* = 0.191, *n* = 48; LDx* Right – pre-SAH median 0.00% [IQR 0.00–0.00%] versus post-SAH median 0.00% [IQR 0.00–0.00%], W = 75.0, *P* = 0.943, *n* = 48.

To assess the proportion of animals exhibiting impaired autoregulation over time, we calculated the percentage of animals exceeding the impairment threshold (PRx* > 0.3 or LDx* > 0.5) at each time point. Only a limited number of animals demonstrated LDx* values exceeding 0.5. These results are plotted in Fig. 4. Across both pre- and post-SAH periods, group-level prevalence of PRx* exceeding the 0.3 cut off remained below the 30% and LDx* exceeding the 0.5 cut off remained below the 20% threshold. PRx* showed a modest increase in the proportion of animals above threshold following SAH induction, while LDx* values from both hemispheres relatively stable when comparing the pre versus post period (see Fig. 4A).

A comparison of the percentage of time spent above threshold before and after SAH is shown in Fig. 4B. For PRx*, animals spent significantly more time above the 0.3 threshold post-SAH [median: 0.0% pre versus 1.9% post, *P* < 0.001, *n* = 45; rank-biserial *r* = 0.091, 95% CI (0.000, 0.216)]. In contrast, no significant changes were observed for LDx* (applying the 0.5 threshold) in either the left hemisphere [pre: 0.00% (IQR 0.00–0.62%), post: 0.00% (IQR 0.00–0.00%), *W* = 70.0, *P* = 0.191, *n* = 48] or the right hemisphere [pre: 0.00% (IQR 0.00–0.00%), post: 0.00% (IQR 0.00–0.00%), *W* = 75.0, *P* = 0.943, *n* = 48]. These findings indicate a slight post-SAH increase in PRx* at the group level (although below the 0.3 threshold), with no consistent differences in LDx* between hemispheres or observed periods.

### Effect of haemorrhage severity

**Figure 5:**
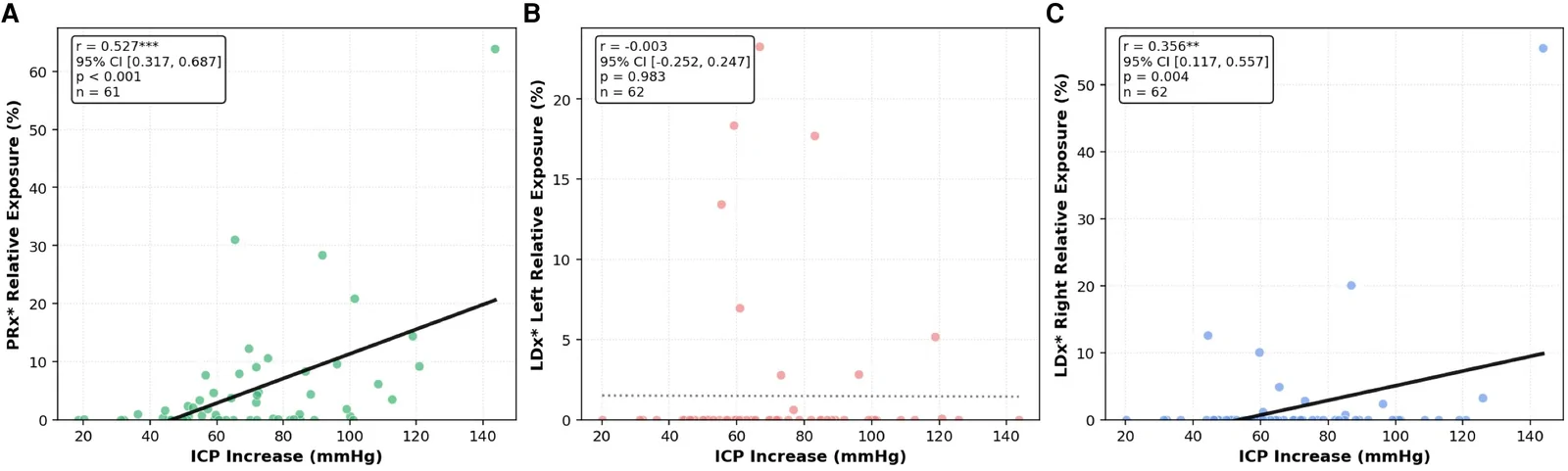
**ICP increase versus relative time (%) spent with impaired cerebral autoregulation.** Scatterplots showing the relationship between peak ICP rise after SAH induction and autoregulation impairment during the post-SAH period (15–60 min). Each data point represents one animal. Pearson correlation coefficients (***r***) w**I**th 95% confidence intervals and *P*-values are shown for each panel. Linear regression line styles indicate statistical significance: solid lines (*P* < 0.05), dashed lines (*P* < 0.1), dotted lines (*P* ≥ 0.1). **(A)** PRx* relative exposure, percentage of time spent with PRx* > 0.3 (*n* = 61). **(B)** LDx* Left relative exposure, percentage of time spent with LDx* > 0.5 on the left (SAH-affected) hemisphere (*n* = 62). **(C)** LDx* Right relative exposure, percentage of time spent with LDx* > 0.5 on the right (contralateral) hemisphere (*n* = 62).

Among the three severity markers assessed, only ICP increase showed significant association with impaired cerebral autoregulation (Fig. 5 and Suppl. Fig. 5), with PRx* relative exposure (Fig. 5A), defined as the percentage of time with PRx* > 0.3, showing positive correlation with the magnitude of ICP elevation [*r* = 0.527, 95% CI (0.317, 0.687), *P* < 0.001, *n* = 61].

This indicates that animals with more pronounced intracranial hypertension following SAH spent a greater proportion of the post-SAH period with impaired pressure reactivity. Relative exposure ranged from 0% to over 60%, although only a small number of animals exhibited high impairment durations; just three exceeded 25%. The positive slope and correlation coefficient are therefore partially influenced by these few high-exposure cases, which likely reflect more severe individual responses to haemorrhage.

In contrast, ICP increase was not significantly associated with time spent with LDx* above 0.5 on the left [Fig. 5B; *r* = −0.003, 95% CI (−0.252, 0.247), *P* = 0.983, *n* = 62]. On the right side, there was a positive correlation between the amount of ICP increase and the percentage of time spend with LDx* above 0.5 [Fig. 5C; *r* = 0.356, 95% CI (0.117, 0.557), *P* = 0.004, *n* = 62].

LDF drop, representing the magnitude of cerebral blood flow reduction during haemorrhage, was also not significantly associated with autoregulation indices. Correlations with time spent with PRx* > 0.3 were weak and non-significant for both LDF drop on the left [Suppl. Fig. 5A; *r* = 0.180, 95% CI (−0.078, 0.415), *P* = 0.169, *n* = 60] and right side [Suppl. Fig. 5B; *r* = 0.154, 95% CI (−0.104, 0.393), *P* = 0.240, *n* = 60]. Similarly, no associations were observed between LDF drop and time spent with LDx* > 0.5 from the left [Suppl. Fig. 5C; *r* = 0.048, 95% CI (−0.204, 0.294), *P* = 0.710, *n* = 62]. A mild association was observed between the extend of drop in LDF after SAH induction and the proportion of time spend with LDx* above 0.5 [Suppl. Fig. 5E; *r* = 0.271, 95% CI (0.023, 0.488), *P* = 0.033, *n* = 62] for the hemispheres.

### Comparison between PRx and LDx

**Figure 6:**
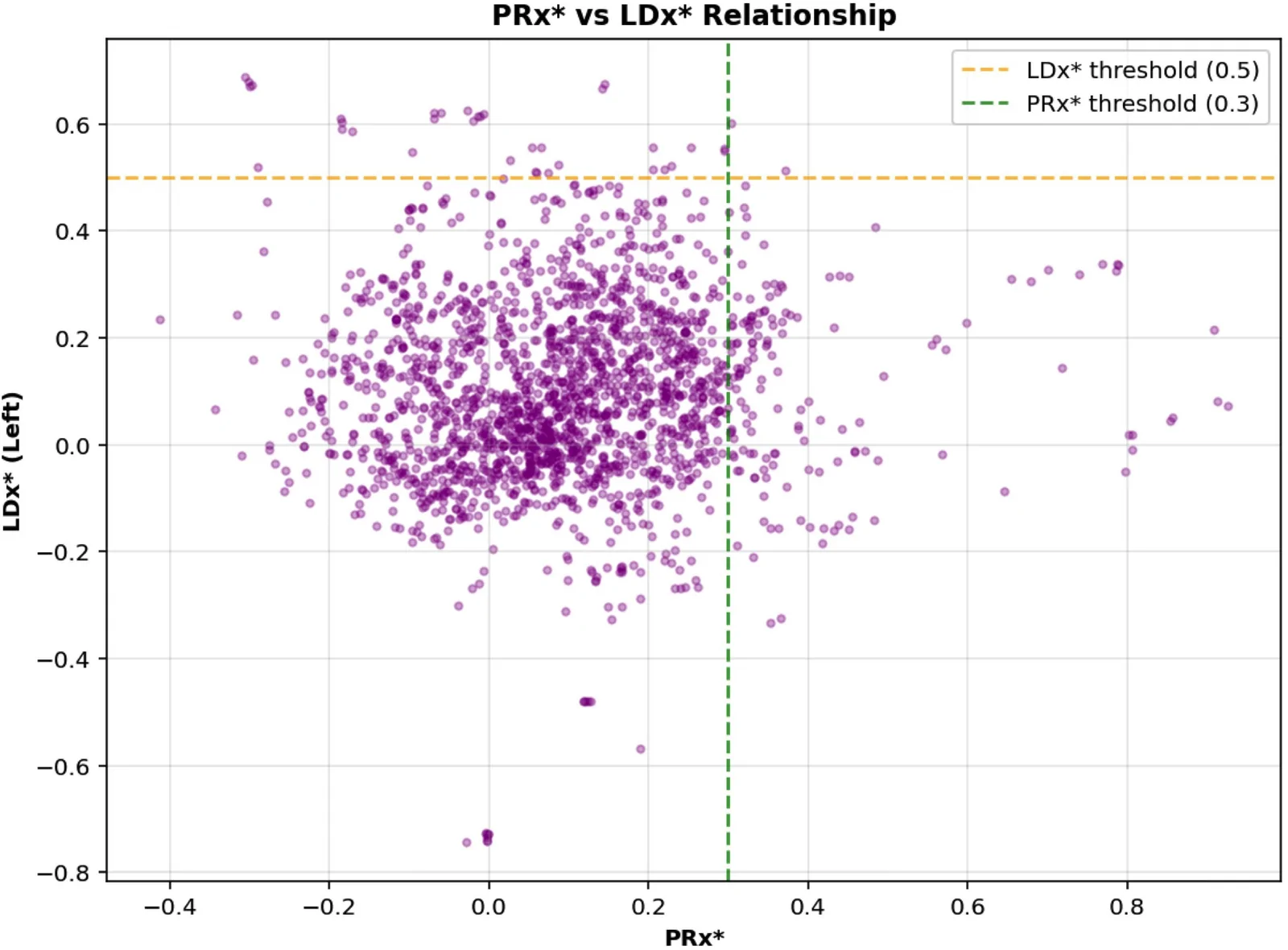
PRx*–LDx* relationship analysis in the left hemisphere following subarachnoid haemorrhage. Scatter plot showing the relationship between PRx* and LDx* (left hemisphere) values during the post-SAH analysis window (15–60 min). The green dashed line indicates the impairment threshold for PRx* (0.3) and the orange dashed line indicates the impairment threshold for LDx* (0.5). Each point represents a paired observation from the combined dataset (*n* = 2000 randomly sampled points drawn from all included animals, for visualization purposes). .

Analysis of 63 animals was used to assess the relationship between PRx* and LDx* during the post-SAH period (Fig. 6). The scatter plot of all paired observations showed substantial variability in the PRx-LDx relationship, with both indices spanning a wide range of negative to positive values. Most observations clustered around 0.1–0.2 for both indices, with considerable scatter and frequent disagreement between the measures. Overall, PRx and LDx* showed a statistically significant but weak positive correlation across all paired observations [*r* = 0.089, 95% CI (0.083, 0.095), *P* < 0.001, *n* = 101 369 observation pairs].

## Discussion

This study characterizes cerebral autoregulation during the hyperacute phase following experimental subarachnoid haemorrhage. Overall, pressure reactivity appeared largely preserved across the study population, with most animals showing PRx* values below the impairment threshold and no consistent positive correlation between arterial blood pressure and intracranial pressure slow changes. Only a small subset of animals demonstrated sustained elevations in PRx* above 0.3, and these cases were predominantly associated with exposure to markedly elevated intracranial pressure. This may be due to CPP dropping and remaining below the lower limit of reactivity, or to induced, transient, vascular paralysis. Flow-based reactivity, as measured by LDx* on both hemispheres, showed minimal deviation from baseline and limited evidence of impairment.

These findings seem to suggest that acute disruption of cerebral autoregulation appears uncommon immediately after SAH induction. This would support the understanding that autoregulatory dysfunction primarily arises from biological processes, such as inflammation, oxidative stress and gene expression changes, which require time to develop. However, the observation that a small number of animals with severe ICP elevations exhibited impaired PRx* raises the possibility that mechanical injury to pressure-sensitive vascular mechanisms may contribute to early dysfunction in select cases.

Studies investigating cerebrovascular CO_2_ reactivity following experimental SAH have yielded variable results across species and models. Deringer *et al*.^26^ found that in dogs, CO_2_ reactivity remained intact despite angiographically confirmed vasospasm following cisternal blood injections, suggesting preserved microvascular responsiveness despite large-vessel narrowing. Similarly, in rabbits, SAH reduced resting CBF and increased cerebrovascular resistance, yet CBF still increased robustly to hypercapnia and hypoxia, indicating preserved vascular reactivity.^27^ Conversely, cats demonstrated blunted hypercapnic CBF responses, particularly at higher PaCO_2_ levels, despite unchanged normocapnic CBF, potentially reflecting combined effects of large-artery vasospasm and impaired small-vessel reactivity.^28^ Rasmussen *et al*.^29^ investigated temporal autoregulation profiles following SAH in rats using ^133^Xenon intracarotid injection to measure hemispheric CBF under controlled blood pressure manipulations. Their findings revealed marked and persistent autoregulation impairment at both 2- and 5-day post-haemorrhage, evidenced by rightward shift and narrowing of the autoregulatory plateau. Importantly, this dysfunction persisted even after angiographic vasospasm resolution, providing early insight that autoregulatory impairment involves microvascular dysregulation rather than solely large-vessel spasm mechanisms.

Conzen-Dilger *et al*.^30^ investigated haemorrhage dynamics’ impact on cerebral autoregulation using PRx, in a cisternal infusion SAH model where blood injection rates were varied. Rapid ICP elevation caused early (1–5 h) autoregulation impairment with persistently elevated PRx values, whereas slower infusion resulted in only transient or no significant disruption. PRx returned to physiological levels in all groups except those with rapid injection. These results suggest that the dynamics of ICP increase, might play a role in cerebrovascular dysfunction after experimental SAH.

Comparing multiple autoregulatory indices, Owen *et al*.^9^ identified that PRx and ORx correlated best with outcome in SAH patients. Both indices demonstrated poor minute-to-minute agreement, but better concordance when averaged over hourly intervals. However, when comparing compiled hourly PRx and ORx data pairs from 76 SAH patients, Kastenholz *et al*.^31^ observed no correlation between both indices. In a piglet model with active perturbations of arterial blood pressure, LDx has been compared to other autoregulatory indices.^24,25^ Compared to PRx, both indices were able to detect the lower limit of autoregulation, assessed by plotting of autoregulatory curves.^24^ PRx demonstrated slightly reduced sensitivity but maintained consistent specificity across different ICP ranges. The authors conclude that while PRx and LDx both provide insight into cerebrovascular autoregulation, they capture different physiological processes. Congruent to our results, as PRx primarily reflects pressure-driven volume changes, whereas LDx more directly relates to local flow-based aspects of autoregulatory control they represent different underlying physiological mechanisms. Therefore, they assess different aspects of cerebral autoregulation, which are not interchangeable.

### Limitations

Several limitations should be acknowledged. First, some uncertainty in the reliability of our data arises from the clustering of PRx* and LDx* values around zero, which may very well be, at least in part, due to the limited variability of ABP within the relatively short analysis windows, and thus poor reliability of the methodology.^32^ Nevertheless, the clear, and expected, increase in PRx* after SAH compared to baseline seems to support the overall validity of our findings, as this response is consistent with impaired cerebral autoregulation in the post-SAH phase.

A further limitation relates to the choice of animal model. The endovascular perforation model used in the parent study was selected for its ability to reproduce the acute haemodynamic consequences of aneurysm rupture, including the sudden ICP surge and transient global cerebral ischaemia. However, as highlighted in a systematic review of preclinical SAH methodology by Grüter *et al*., the endovascular perforation model carries inherent operator-dependent variability in haemorrhage severity, which may have contributed to the wide inter-individual variation in our severity markers.^33^ Alternative models such as the rabbit blood-shunt model described by Marbacher *et al*.^34^ were specifically engineered to reproduce hyperacute ICP dynamics with higher reproducibility and reduced examiner-dependent variability, and may be better suited for dedicated studies of hyperacute cerebral autoregulation. During the project, different filaments were used for perforation introducing variability in the model. Filament size data were not available in the high-frequency physiological datasets used for the present analysis, precluding formal assessment of filament size as a potential confounder of haemorrhage severity and autoregulation indices.

This study was conducted exclusively in male rats, which is common practice in experimental SAH research for practical and husbandry-related reasons. However, given known sex-related differences in cerebrovascular physiology, autoregulatory function and SAH epidemiology, the generalizability of these findings to female animals and to the broader human population, in which SAH disproportionately affects women, cannot be assumed.

Furthermore, there is only limited evidence available on the use of autoregulatory indices in rodent models of SAH or traumatic brain injury, and the transmission dynamics of ABP and intracranial volume changes in rats remain insufficiently characterized. To our knowledge, no previous study has validated this methodology in a rodents SAH perforation model. However, the observed direction of PRx* changes following SAH aligns with expectations based on impaired autoregulation, which increases confidence in our approach.

As this study represents a secondary analysis of data originally collected to investigate neuroprotective treatments initiated 60 min after SAH induction, no formal a priori sample size calculation was performed for the present research question, and findings should be considered exploratory and warrant cautious interpretation. Rather than performing a post hoc power analysis, which is circular as statistical power is mathematically determined by the observed effect size and sample size, we report effect sizes with 95% confidence intervals for all primary comparisons. It should be noted that the rank-biserial correlation has known limitations when a large proportion of observations are tied at zero, as was the case for LDx* exposure, where the estimate may be inflated and should be interpreted with caution. Future confirmatory experiments in rodent models should incorporate induced ABP variability to better assess the robustness and validity of autoregulatory indices such as PRx* and LDx*.

## Conclusion

This study provides insights into cerebral autoregulation in the hyperacute phase following experimental subarachnoid haemorrhage. While population-averaged measures suggested preserved autoregulatory function, a small subset of animals exhibited early impairment, particularly in relation to elevated intracranial pressure. PRx* and LDx* demonstrated only moderate correlation and frequent discordance, suggesting they capture different aspects of autoregulatory physiology. These findings highlight heterogeneity of the autoregulatory responses immediately after haemorrhage and support the use of complementary monitoring approaches to better understand early cerebrovascular dysfunction.

## Supplementary Material

fcag356_Supplementary_Data

## Data Availability

The data used in this analysis are available from the authors upon reasonable request for use by qualified researchers.
