# Supplementary material for: Cerebral autoregulation in the hyperacute phase after experimental subarachnoid haemorrhage using an endovascular perforation model
Source: Brain Commun. 2026 Sep 16;8(5):fcag356. doi: 10.1093/braincomms/fcag356 (PMC13599029; doi:10.1093/braincomms/fcag356)
Supplement: Supplementary file 1 [file fcag356_supplementary_data.docx]

| **Variable** | **Value** |
| --- | --- |
| Sample size | n = 69 |
| ICP Pre (mmHg) | 7.63 [5.1-9.8] |
| ICP Peak (mmHg) | 64.3 [50.3 to 83.0] |
| ICP Post (mmHg) | 15.24 [11.6-19.5] |
| ABP Pre (mmHg)^#^ | 125.90 [103.4-146.7] |
| ABP Post (mmHg)^#^ | 107.10 [96.52-122.10] |
| LDF Left Pre (PU) | 153.50 [96.5-261.5] |
| LDF Left Post (PU) | 82.90 [38.4-145.9] |
| LDF Right Pre (PU) | 256.70 [155.2-392.8] |
| LDF Right Post (PU) | 195.70 [107.0-300.7] |
| LDF Left Drop (PU) | 96.5 [69.9 to 166.8] |
| LDF Right Drop (PU) | 135.5 [76.3 to 213.7] |
| Sugawara Grading | 10.0 [7.0 to 14.0] |
| PRx* (Pre) | 0.015 [-0.021 to 0.060] |
| PRx* (Post) | 0.062 [-0.002 to 0.149] |
| LDx* Left (Pre) | 0.097 [-0.016 to 0.186] |
| LDx* Left (Post) | 0.121 [0.037 to 0.172] |
| LDx* Right (Pre) | 0.111 [0.046 to 0.224] |
| LDx* Right (Post) | 0.124 [0.054 to 0.192] |

**Suppl. Table 1. Summary statistics of pre- and post- SAH induction physiological measurements and derivatives expressed as medians with Q1 to Q3.**

^#^ Due to calibration issues the absolute blood pressure values might underestimate the actual true values as some animals started off with negative pressures. This, however does not affect the calculation of derivatives such as the pressure reactivity index.

**Supplemental Figures**

Supplemental Figure 1

****Suppl. Fig. 1.** (**A-D**)** Pressure Reactivity Index (PRx) calculated using different time windows in sham animals. Left panels show PRx over time during the 90-minute period with highest data availability for each animal. Green lines represent mean PRx values across all animals (n=21), with shaded areas indicating ±1 standard deviation. Right panels display boxplots summarizing the distribution of all PRx values within each 90-minute window. Red diamonds indicate mean values, with mean and standard deviation displayed in text boxes. Red dashed lines mark the PRx = 0.3 threshold indicating impaired cerebral autoregulation occurring above this value. From top to bottom: (A) PRx calculated using 30-second, (B) 60-second, (C) 120-second, (D) and 300-second averaging windows. Longer calculation windows progressively reduce variability while maintaining similar mean values, demonstrating that window length affects temporal resolution but not overall PRx magnitude in this population.

Supplemental Figure 2

Suppl. Fig. 2. (A-D) Laser Doppler Flowmetry Reactivity Index (LDx) for left and right hemispheres calculated using different time windows in sham animals. Left panels show LDx over time during the 90-minute period with highest data availability for each animal. Red lines represent mean LDx values for the left hemisphere, blue lines for the right hemisphere, across all animals (n=21), with corresponding-colored shaded areas indicating ±1 standard deviation. Right panels display paired boxplots summarizing the distribution of all LDx values within each 90-minute window for left (red) and right (blue) hemispheres. Colored diamonds indicate mean values, with hemisphere-specific means and standard deviations displayed in text boxes. Gray dashed lines mark the LDx = 0.5 threshold indicating impaired cerebrovascular reactivity. From top to bottom: (A) LDx calculated using 30-second, (B) 60-second, (C) 120-second, and (D) 300-second averaging windows. Both hemispheres show similar patterns, with longer calculation windows progressively reducing variability while maintaining comparable mean values between sides, demonstrating bilateral symmetry in cerebrovascular reactivity responses.

Supplemental Figure 3

Suppl. Fig. 3. CPP standard deviation differences across multiple window sizes in the pre- and post-SAH periods

Boxplots show the mean absolute difference of the sliding window standard deviation of cerebral perfusion pressure (CPP), computed across window sizes of 0.5, 1.0, and 5.0 minutes. Each data point represents an individual animal's average |Δ Std| value for a given window size and period. Blue boxes correspond to the pre-SAH period; orange boxes correspond to the post-SAH period. The number of animals contributing to each box was: 0.5-minute window, n = 64 (pre-SAH) and n = 66 (post-SAH); 1.0-minute window, n = 64 (pre-SAH) and n = 64 (post-SAH); 5.0-minute window, n = 53 (pre-SAH) and n = 56 (post-SAH).

Shorter windows (e.g., 30 seconds) exhibit higher variability in standard deviation, reflecting greater sensitivity to transient fluctuations. As window size increases, |Δ Std| values decrease markedly, indicating greater stability in the CPP signal. Across all window sizes, pre-SAH data showed slightly greater variability than post-SAH data. These results support the use of a 5-minute window or 300 seconds (standard for PRx calculation), which demonstrates high CPP signal stability consistent with stationarity assumptions.

Supplemental Figure 4

Suppl. Fig. 4. (A-F) ABP and ICP Variability Within 5-Minute PRx Calculation Windows***

Analysis of physiological signal variability within sliding 5-minute windows used for PRx* calculation. **(A-B)** Histograms showing ABP standard deviation decreased significantly post-SAH (median: 1.3→0.6 mmHg, p<0.001) while ICP standard deviation remained stable (0.35→0.33 mmHg, p<0.001) (Mann-Whitney U test). **(C)** Positive correlation (Pearson correlation) between ABP variability and mean PRx* post-SAH (r=0.450, p<0.001); dashed line indicates impairment threshold (PRx*>0.3). **(D-E)** Box plots confirming significant pre-vs-post differences (Wilcoxon signed-rank test). **(F)** ABP coefficient of variation showing relative variability patterns. n=52 animals pre-SAH, n=60 post-SAH. This demonstrates sufficient signal variation exists for valid PRx* calculation despite reduced ABP fluctuations following hemorrhage.

Supplemental Figure 5

**Suppl. Fig. 5. (A-F) LDF drop versus relative time (%) spent with impaired cerebral autoregulation.**

Scatterplots showing the relationship between the magnitude of cerebral blood flow reduction during SAH induction (LDF drop) and autoregulation impairment during the post-SAH period (15 to 60 minutes). Each data point represents one animal.

**(A-B)** PRx* relative exposure, percentage of time spent with PRx* > 0.3, plotted against LDF Left Drop (A, n = 60) and LDF Right Drop (B, n = 60).

**(C-D)** LDx* Left relative exposure, percentage of time spent with LDx* > 0.5 on the left (SAH-affected) hemisphere, plotted against LDF Left Drop (C, n = 62) and LDF Right Drop (D, n = 62).

**(E-F)** LDx* Right relative exposure, percentage of time spent with LDx* > 0.5 on the right (contralateral) hemisphere, plotted against LDF Left Drop (E, n = 62) and LDF Right Drop (F, n = 62). LDF Left Drop = reduction in laser Doppler flowmetry perfusion units (PU) on the left (SAH-affected) side; LDF Right Drop = reduction on the right (contralateral) side. Pearson correlation coefficients (r) with 95% confidence intervals and p-values are shown for each panel. Linear regression line styles indicate statistical significance: solid lines (p < 0.05), dashed lines (p < 0.1), dotted lines (p >= 0.1).
